# Targeting the Hyperglycemic Pre‐Metastatic Niche to Prevent Breast Cancer Bone Metastasis: Mechanisms and Therapeutic Strategies

**DOI:** 10.1002/advs.202519858

**Published:** 2025-11-16

**Authors:** DuJiang Yang, Jiexiang Yang, GuoYou Wang

**Affiliations:** ^1^ The Affiliated Traditional Chinese Medicine Hospital ,Southwest Medical University NO. 182, Chunhui Road, Longmatan District Luzhou Sichuan 646000 P. R. China; ^2^ Center for Orthopedic Diseases Research The Affiliated Traditional Chinese Medicine Hospital Southwest Medical University NO. 182, Chunhui Road, Longmatan District Luzhou Sichuan 646000 P. R. China

**Keywords:** breast cancer bone metastasis, glucose deprivation, hyperglycemia, pre‐metastatic niche, RAGE inhibition, therapeutic targeting, tumor microenvironment

## Abstract

The recent pioneering study by Ye et al. (Adv Sci. 2025;12(37):e0504924) provides compelling evidence that systemic hyperglycemia orchestrates a pre‐metastatic niche (PMN) in bone, promoting breast cancer metastasis and identifying Receptor for Advanced Glycation End‐products (RAGE) inhibition as a viable therapeutic strategy. While this work represents a significant conceptual advance by linking systemic metabolism to organotropic metastasis, the analysis identifies several substantive challenges that must be addressed to translate this finding into clinical practice. First, the authors clarify that the primary driver of enhanced bone metabolism is likely tumor‐derived educative signals, with hyperglycemia acting as a secondary sensitizer rather than a sole instigator; this hierarchical causality requires further dissection. Second, the pleiotropic roles of RAGE in physiology and immunity raise concerns about the potential for on‐target, adverse effects with long‐term inhibition, necessitating the development of more targeted approaches. Finally, while the primary metabolic therapy proposed is GP‐mediated glucose deprivation, the therapeutic efficacy demonstrated in a preventative model requires validation in scenarios of established micro‐metastases, which mirror the clinical reality more closely. This critical appraisal underscores the need for a more nuanced understanding of the niche's biology and a rigorous evaluation of the proposed therapeutic paradigm's safety and timing.

**To the Editor,**

We read with great interest the seminal study by Ye et al.,^[^ ^1^ ^]^ which identifies a hyperglycemia‐associated pre‐metastatic niche (PMN) in bone and proposes RAGE inhibition as one potential therapeutic strategy against breast cancer bone metastasis. The authors elegantly demonstrate how systemic hyperglycemia remodels the bone marrow stroma to facilitate metastatic seeding, thereby integrating metabolic dysregulation with the classical “seed and soil” hypothesis—a notable conceptual advance. While we appreciate the robustness of their findings, several aspects of the proposed mechanism and its translational applicability merit further nuanced discussion.^[^ ^1^ ^]^

First, regarding the formation of the “hyperglycemic PMN,” the authors clarify in their response that the primary driver of enhanced glucose metabolism in bone is likely the direct regulatory influence of breast cancer cells—via exosomes or other signaling molecules—with systemic hyperglycemia serving a secondary, supportive role. This important distinction should be emphasized in interpreting the PMN mechanism. Indeed, the study does not fully decouple the contributory effects of tumor‐derived educative factors (e.g., VEGF, TGF‐β) from the direct metabolic impact of high glucose. To better resolve this hierarchy, future work could profile educative factors in the plasma of tumor‐bearing hyperglycemic mice or examine whether conditioned media from breast cancer cells can recapitulate PMN features in non‐tumor‐bearing hyperglycemic hosts. Such experiments would help clarify whether hyperglycemia acts predominantly as a sensitizer that amplifies tumor‐derived signals—a nuance with implications for identifying which patients may benefit from metabolic interventions.

Second, while the study explores Receptor for Advanced Glycation End‐products (RAGE) blockade as a therapeutic angle, the authors note that their central metabolic strategy relies on the glucose‐depriving effect of the engineered enzyme Glycolytic Pathway (GP) to induce tumor starvation. This point is well‐taken. Nonetheless, the discussion of RAGE inhibition remains highly relevant from a translational perspective.RAGE is a multifunctional pattern recognition receptor involved not only in pathology but also in physiological processes such as tissue repair and immune regulation.^[^ ^2^, ^3^ ^]^ Long‐term systemic inhibition may therefore carry risks of on‐target, off‐tissue effects—a concern not addressed in the current study. Moreover, the potential for compensatory activation of alternative pro‐metastatic pathways upon RAGE blockade remains unexplored. We concur that future efforts should prioritize the development of tissue‐specific RAGE antagonists or ligand‐specific blockers to minimize systemic consequences while preserving therapeutic efficacy.^[^ ^3^, ^4^ ^]^

Finally, the therapeutic efficacy demonstrated in a preventive model—where treatment begins at or before tumor inoculation—requires validation in more clinically relevant settings. Most patients present with established micro‐metastases or are at high risk of recurrence, rather than at the initial stage of seeding. It will be essential to test whether RAGE inhibition or GP‐based metabolic therapy can reverse an already‐established PMN or suppress the outgrowth of existing micro‐metastases. Combining these approaches with standard bone‐targeting agents (e.g., bisphosphonates or denosumab) represents a logical next step,^[^ ^4^, ^5^ ^]^ though the potential for synergistic toxicity—such as osteonecrosis—must be carefully evaluated. Studies initiating treatment after the detection of circulating tumor cells or micro‐metastases would offer a more realistic assessment of the therapeutic window.

In summary, the work by Ye et al. represents a significant step forward in bridging systemic metabolism with metastasis research. Their findings open important avenues for therapeutic exploration, particularly through GP‐mediated glucose deprivation. Moving forward, we encourage a deeper mechanistic dissection of the tumor‐metabolism interplay, along with rigorous safety and efficacy testing of anti‐RAGE and metabolic strategies in advanced, clinically aligned disease models.

Sincerely,

**DuJiang Yang, MD**

**Guoyou Wang, MD, PhD**

**President and Party Secretary of the Southwest Medical University Hospital of traditional Chinese medicine**

## Conflict of Interest

The authors declare no conflict of interest.
